# Potential masking of new-onset or relapsed eosinophilic granulomatosis with polyangiitis during benralizumab treatment: A case series

**DOI:** 10.1016/j.jacig.2025.100551

**Published:** 2025-08-07

**Authors:** Shin-ichiro Ohmura, Haruka Yonezawa, Yusuke Ohkubo

**Affiliations:** Department of Rheumatology, Seirei Hamamatsu General Hospital, Shizuoka, Japan

**Keywords:** Benralizumab, eosinophilic granulomatosis with polyangiitis, eosinophilia

## Abstract

Eosinophilic granulomatosis with polyangiitis can manifest without eosinophilia and eosinophilic infiltration during benralizumab treatment, emphasizing the need for clinical vigilance beyond eosinophil counts.

Eosinophilic granulomatosis with polyangiitis (EGPA) is a rare disease characterized by necrotizing vasculitis in small vessels with eosinophilic inflammation. Benralizumab, an anti–IL-5 receptor mAb, demonstrated a high remission rate and significantly reduced eosinophil counts.^1^^,^^2^ However, EGPA relapsed even if serum eosinophil count was normal, and anti–IL-5 therapy may not be fully effective on EGPA manifestations.^3^, ^4^, ^5^ In this case series, we present 2 cases of new-onset and relapsed EGPA without eosinophilia and eosinophilic infiltration under benralizumab treatment, indicating that benralizumab may have potential masking effects by depleting eosinophil counts in patients with EGPA.

## Patient 1

**Fig 1:**
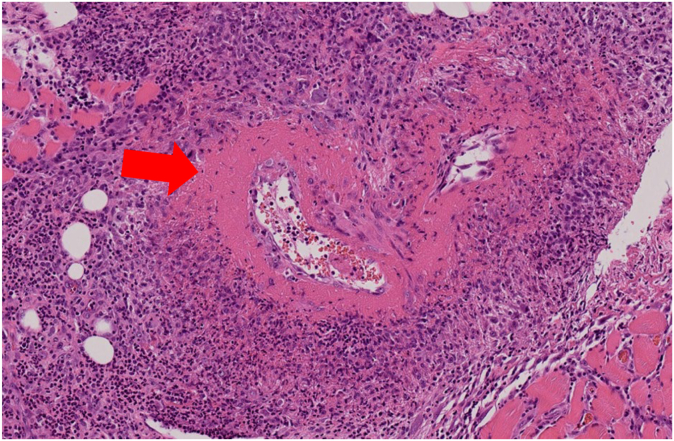
Pathologic findings in patient 1 under benralizumab treatment. Pathologic findings revealed fibrinoid necrosis but no granuloma or eosinophil infiltration in patient 1.

A 65-year-old woman was referred to our department with intractable myalgia and paresthesia in the lower extremities. She had bronchial asthma, sinusitis, and eosinophilic pneumonia, and she was treated with prednisolone. Before treatment, her serum eosinophil count was 3142/μL. However, her condition was intractable and benralizumab administration was initiated 20 months before consultation. Before benralizumab introduction, her serum eosinophil count was 420/μL. After the treatment, her serum eosinophil count was 0/μL and her prednisolone was discontinued after 13 months of benralizumab treatment. However, multiple bilateral pulmonary nodular shadows, nasal polyps, and worsening sinusitis appeared despite the fact that her serum eosinophil count 6 months before the consultation was zero. In addition, general malaise, myalgia, and paresthesia in the patient’s legs developed 1 month before consultation. Thus, she was referred to our department.^4^ On admission, her body temperature was 38.0°C and her body weight was 6 kg less than 6 months before. Physical examination showed grasping pain in the lower extremities and bilateral sensory deficits in the feet. Her blood test results were as follows: white blood cell count, 4520/μL; eosinophil count, 0/μL; lactate dehydrogenase level, 213 U/L; creatine kinase level, 63 U/L; creatinine level, 0.57 mg/dL; C-reactive protein level, 1.84 mg/dL; and IgE level, 750.2 U/mL. The results of testing for myeloperoxidase–antineutrophil cytoplasmic antibody (ANCA) and proteinase 3 (PR3)-ANCA were negative. Whole-body computed tomography showed sinusitis and multiple nodular shadows in both lungs. Magnetic resonance imaging of the lower legs showed a diffuse high-intensity area. Nerve conduction studies showed sensory neuropathy. Muscle biopsy revealed fibrinoid necrosis but no granuloma or eosinophil infiltration (Fig 1). Despite the lack of eosinophilia and eosinophilic filtration under benralizumab treatment, the patient had severe allergic disease with an increasing serum eosinophil count and no granulomatosis with polyangiitis surrogate marker, including PR3-ANCA, abnormal urinalysis findings, and granulomas with pathologic findings. Thus, she was diagnosed with EGPA without eosinophilia and eosinophilic filtration under benralizumab treatment based on the classification of primary systemic vasculitis and the 2022 American College of Rheumatology/European Alliance of Associations for Rheumatology classification criteria for EGPA.^6^ The patient’s Birmingham Vasculitis Activity Score was 17 points. She was treated with high-dose prednisolone and intravenous cyclophosphamide. Her condition improved after treatment; however, her eosinophilic pneumonia and neuropathy relapsed when her prednisolone dose was 15 mg per day. In addition, her serum eosinophil count increased to 2346/μL. She was treated again with high-dose prednisolone for relapse of EGPA.

## Patient 2

**Fig 2:**
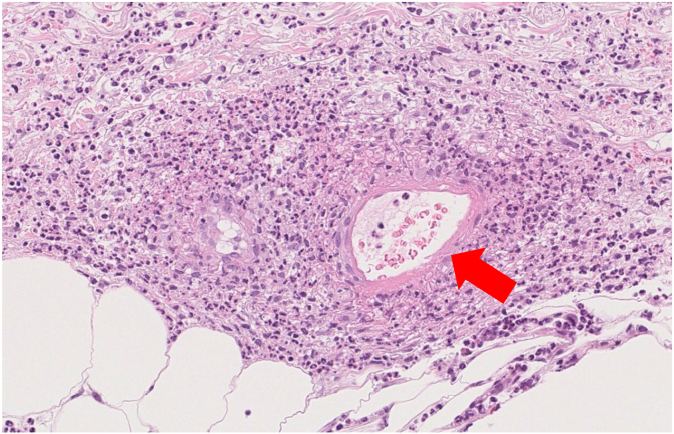
Pathologic findings in patient 2 under benralizumab treatment. Pathologic findings revealed necrotizing vasculitis but no granuloma or eosinophil infiltration in patient 2.

A 77-year-old woman was referred to our department with purpura and numbness in her legs. She had a 10-year history of intractable bronchial asthma and sinusitis. Two years before admission, she was diagnosed with EGPA based on the 2022 American College of Rheumatology/European Alliance of Associations for Rheumatology classification criteria for EGPA and treated with high-dose prednisolone.^6^ At the time of EGPA diagnosis, her serum eosinophil count was 4360/μL, and skin biopsy showed eosinophilic infiltration (see Fig E1 in the Online Repository at www.jaci-global.org). However, her condition relapsed frequently, and as a result, benralizumab was introduced for refractory EGPA 3 months before her admission. After initiation of benralizumab therapy, her condition remained stable and her serum eosinophil counts were zero. However, her purpura and neuropathy relapsed without eosinophilia after 3 months of benralizumab administration. On admission, her vital signs were normal, with no weight loss. Physical examination revealed purpura on her lower legs; right toe extension was weak with Manual Muscle Testing 2. Additionally, the patient experienced decreased sensations of touch as well as thermal and pain in the right foot. Blood examination showed a white blood cell count of 3630/μL, eosinophil count of 0/μL, albumin level of 2.9 g/dL, lactate dehydrogenase level of 264 IU/L, creatinine level of 0.57 mg/dL, creatine kinase level of 20 U/L, C-reactive protein level of 2.27 mg/dL, and IgE level of 4540 U/mL. The results of testing for myeloperoxidase-ANCA and PR3-ANCA were negative. Whole-body computed tomography scans showed no abnormal findings. Nerve conduction studies showed sensory neuropathy. Skin biopsy showed necrotizing vasculitis without granuloma or eosinophil infiltration (Fig 2). The patient was diagnosed with relapse of EGPA without eosinophilia and eosinophilic infiltration under benralizumab treatment. Her Birmingham Vasculitis Activity score was 11 points. She was treated with high-dose prednisolone and mepolizumab.

EGPA is a T_H_2 cell–mediated disease, and the upregulation of IL-5 and IL-4/IL-13 pathways is related to EGPA development.^7^^,^^8^ On the other hand, EGPA is also associated with T_H_1 cell– and T_H_17 cell–associated cytokines.^8^ Notably, T_H_17 cell levels increase when EGPA is in the active phase, and this increase is related to EGPA recurrence.^8^ Thus, anti–IL-5 therapy alone may not be fully effective on EGPA manifestations because the treatment untreated the T_H_1 cell and T_H_17 cell arms of the immune system.^3^ A previous study showed that in a patient with EGPA, new lesions appeared even in normal peripheral blood eosinophils under IL-5 treatment, improving after high-dose glucocorticoid treatment and decreasing serum IL-17 level.^3^ Another report also showed new-onset necrotizing vasculitis without eosinophilia and eosinophilic infiltration during benralizumab treatment.^9^ With regard to our patients, they did not show eosinophilia or eosinophilic infiltration; however, vasculitis symptoms developed in both patients (see Table E1 in the Online Repository at www.jaci-global.org), which is consistent with the findings of previous reports.^3^^,^^9^

On the other hand, pathologic eosinophilic changes were observed in a patient with EGPA under mepolizumab treatment.^5^ A previous study showed that the eosinophil-depleting effect in benralizumab treatment was higher than that in mepolizumab treatment^1^; thus, in addition to serum normal eosinophil count, pathologic eosinophilic changes might not be observed in patients with EGPA under benralizumab treatment.

Although difficult to conclude from our 2 cases, benralizumab may mask new-onset EGPA or EGPA recurrence by depleting eosinophil counts.

In conclusion, physicians should pay attention to vasculitis symptoms during benralizumab treatment in patients with EGPA even if there is no eosinophilic change.

Patient consent: Signed informed consent was obtained from the patients regarding use of the patients’ information for purposes of writing a case report publication.

Data availability statement: The data underlying this article will be shared on reasonable request to the corresponding author.

## Disclosure statement

Disclosure of potential conflict of interest: The authors declare that they have no relevant conflicts of interest.

## Supplementary data

Supplementary Data

Supplementary Fig E1

Supplementary Table E1
