# Supplementary figures and images for: Potential masking of new-onset or relapsed eosinophilic granulomatosis with polyangiitis during benralizumab treatment: A case series
Source: J Allergy Clin Immunol Glob. 2025 Aug 7;4(4):100551. doi: 10.1016/j.jacig.2025.100551 (PMC12396454; doi:10.1016/j.jacig.2025.100551)

## Slide 1
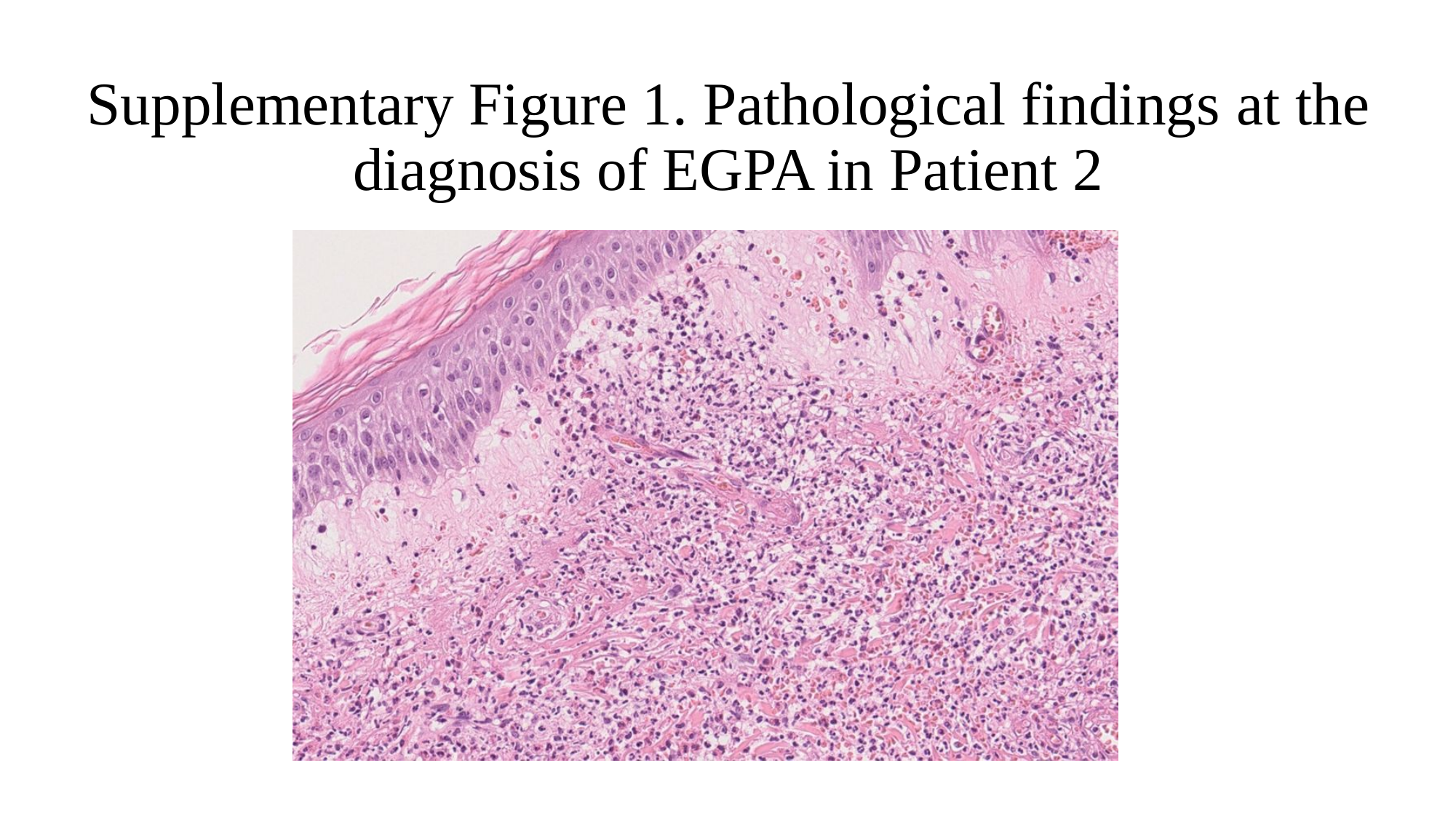

# Supplementary Figure 1. Pathological findings at the diagnosis of EGPA in Patient 2

Supplement: Supplementary Data [file mmc1.pptx]
