# Supplementary material for: Potential masking of new-onset or relapsed eosinophilic granulomatosis with polyangiitis during benralizumab treatment: A case series
Source: J Allergy Clin Immunol Glob. 2025 Aug 7;4(4):100551. doi: 10.1016/j.jacig.2025.100551 (PMC12396454; doi:10.1016/j.jacig.2025.100551)
Supplement: Supplementary Fig E1 [file mmc2.docx]

**Supplementary** **Figure 1.** Pathological findings at EGPA diagnosis in Patient 2.

Pathological findings revealed eosinophilic infiltration in Patient 2.
