# Supplementary material for: Potential masking of new-onset or relapsed eosinophilic granulomatosis with polyangiitis during benralizumab treatment: A case series
Source: J Allergy Clin Immunol Glob. 2025 Aug 7;4(4):100551. doi: 10.1016/j.jacig.2025.100551 (PMC12396454; doi:10.1016/j.jacig.2025.100551)
Supplement: Supplementary Table E1 [file mmc3.docx]

**Supplementary Table 1. New-onset or relapsed EGPA without eosinophilia and eosinophilic infiltration under benralizumab treatment**

| Characteristics | Patient 1 | Patient 2 |
| --- | --- | --- |
| New-onset/relapse | New-onset | Relapse |
| Age (years old) | 65 | 77 |
| Sex | F | F |
| Allergic diseases | Asthma | Asthma |
|  | Sinusitis | Sinusitis |
|  | EP |  |
| Duration of benralizumab (month) | 20 | 3 |
| GC dose at new-onset or relapse of EGPA | None | PSL 6 mg |
| CRP (mg/dL) | 1.84 | 2.27 |
| ANCA-positivity | Negative | Negative |
| Serum eosinophil count at without benralizumab treatment (/μL) | 3142 | 4360 |
| Serum eosinophil count at under benralizumab treatment (/μL) | 0 | 0 |
| Pathological findings at the new-onset or relapse of EGPA | Necrotizing vasculitis | Necrotizing vasculitis |
|  | No eosinophilia | No eosinophilia |
|  | No granulomas | No granulomas |
|  |  |  |
| Visceral involvement | Muscle | Skin |
|  | Lung | Nerve |
|  | Sinus |  |
|  | Nerve |  |
| BVAS | 17 | 11 |
| Treatment | IVMP, GC, IVCY | GC, mepolizumab |

Abbreviations: ANCA, antineutrophil cytoplasmic antibody; BVAS, Birmingham Vasculitis Assessment Score; CRP, C-reactive protein; EGPA, eosinophilic granulomatosis with polyangiitis; EP, eosinophilic pneumonia; F, female; GC, glucocorticoid; IVCY, intravenous cyclophosphamide; IVMP, intravenous methylprednisolone; PSL, prednisolone
